# Lipidomics profiling reveals the role of glycerophospholipid metabolism in psoriasis

**DOI:** 10.1093/gigascience/gix087

**Published:** 2017-09-05

**Authors:** Chunwei Zeng, Bo Wen, Guixue Hou, Li Lei, Zhanlong Mei, Xuekun Jia, Xiaomin Chen, Wu Zhu, Jie Li, Yehong Kuang, Weiqi Zeng, Juan Su, Siqi Liu, Cong Peng, Xiang Chen

**Affiliations:** 1 Department of Dermatology, Xiangya Hospital, Central South University, Xiangya Road #87 Changsha, Hunan, China, 410008; 2 BGI-Shenzhen, Beishan Industrial Zone, Yantian District, Shenzhen, China, 518083; 3 Hunan Key Laboratory of Skin Cancer and Psoriasis, Xiangya Hospital, Central South University, Xiangya Road #87 Changsha, Hunan, China, 410008; 4 China National GeneBank-Shenzhen, Jinsha Road, Dapeng District, Shenzhen, China, 518083

**Keywords:** Psoriasis, Metabolomics, Lipidomics, Glycerophospholipid, MS/MS

## Abstract

Psoriasis is a common and chronic inflammatory skin disease that is complicated by gene–environment interactions. Although genomic, transcriptomic, and proteomic analyses have been performed to investigate the pathogenesis of psoriasis, the role of metabolites in psoriasis, particularly of lipids, remains unclear. Lipids not only comprise the bulk of the cellular membrane bilayers but also regulate a variety of biological processes such as cell proliferation, apoptosis, immunity, angiogenesis, and inflammation. In this study, an untargeted lipidomics approach was used to study the lipid profiles in psoriasis and to identify lipid metabolite signatures for psoriasis through ultra-performance liquid chromatography-tandem quadrupole mass spectrometry. Plasma samples from 90 participants (45 healthy and 45 psoriasis patients) were collected and analyzed. Statistical analysis was applied to find different metabolites between the disease and healthy groups. In addition, enzyme-linked immunosorbent assay was performed to validate differentially expressed lipids in psoriatic patient plasma. Finally, we identified differential expression of several lipids including lysophosphatidic acid (LPA), lysophosphatidylcholine (LysoPC), phosphatidylinositol (PI), phosphatidylcholine (PC), and phosphatidic acid (PA); among these metabolites, LPA, LysoPC, and PA were significantly increased, while PC and PI were down-regulated in psoriasis patients. We found that elements of glycerophospholipid metabolism such as LPA, LysoPC, PA, PI, and PC were significantly altered in the plasma of psoriatic patients; this study characterizes the circulating lipids in psoriatic patients and provides novel insight into the role of lipids in psoriasis.

## Background

Psoriasis is a common and chronic inflammatory skin disease [1, 2]. Histologically, psoriasis is defined by epidermal hyperplasia, keratinocyte differentiation with regenerative maturation, prominent blood vessels in the dermis, and inflammatory leukocyte infiltration. There are 5 main types of psoriasis, i.e., plaque, guttate, inverse, pustular, and erythrodermic. Plaque psoriasis is the most common type (90%) and presents as red and white scaly patches on the top layer of the skin.

Epidemiological investigation has indicated that the incidence of psoriasis in European populations is approximately 2–3% [3–5], whereas in China it is approximately 0.47%; based on this figure, there are approximately 8 million psoriatic patients in China. Although the precise causes of psoriasis are not fully understood, the disease is thought to have a genetic basis that is further complicated by gene–environment interactions [1, 6]

Evidence has shown that abnormal immune responses, particularly in CD4(+) cells, and keratinocyte hyperplasia play critical roles in the pathogenesis of psoriasis. These 2 factors affect each other, forming a positive feedback loop and causing cascading effects. Recently, studies in mouse models as well as clinical studies in humans have demonstrated that the interleukin (IL)-23/IL-17/IL-22 axes are pivotal signaling pathways in psoriasis [7–9].

Lipids have key functions in maintaining normal physiological cellular functions and are believed to be as important as proteins and genes [10]. Currently, there are approximately 10 000 different documented lipids and approximately 600 distinct molecular species of human plasma lipids [11, 12]. Abnormal lipid metabolism is involved in the pathogenesis of several human diseases, such as diabetes, obesity, cancer, and Alzheimer's disease [13, 14]. Lipidomics analyses of both whole plasma and lipoprotein subfractions are essential for current initiatives seeking to better understand the relationships between the composition and function of lipoproteins, and how they are affected by diseases and treatments.

Lipidomics focuses on the structure and function of the complete set of lipids (i.e., the lipidome) produced in a given cell or organism, as well as their interactions with other lipids, proteins, and metabolites. Previously, genomic, transcriptomic, and proteomic analyses had been performed to study psoriasis [15–17]. In the present study, an untargeted lipidomics approach was used to investigate the alteration of lipid metabolites in psoriasis, and the lipid metabolite signature for psoriasis was identified based on ultra-performance liquid chromatography-tandem quadrupole mass spectrometry (UPLC-MS/MS), a highly sensitive and high-resolution method for analyzing complex biological samples. Using this approach to characterize circulating lipids in patients with psoriasis, we found abnormal aspects of lipid metabolism in psoriasis, such as glycerophospholipid metabolism, which provides novel insight into the role of lipids in psoriasis.

## Data Description

Ninety human plasma samples were collected and analyzed in this research to study lipid profiles in psoriasis. Table 1 shows the characteristics of 45 psoriasis and 45 healthy subjects (Additional file 1). UPLC-MS technology was used to detect lipids. Quality control (QC) samples pooled mixtures of all samples were injected among the samples and used to evaluate the experimental quality. Features extracted from all raw data were subjected to data processing, and the features that did not pass quality control were filtered. After data processing, univariate and multivariate statistical analysis were conducted to screen out the significant differentially expressed features. Those features were then identified by searching LipidMaps [12] and the HMDB database [18] and matching standards and targeted data-dependent acquisition (DDA) spectra. Enzyme-linked immunosorbent assay (ELISA) was performed, and the results were used as confirmation and to supplement the lipidomics study.

**Table 1:** Demographics of the study cohort

	Healthy (n = 45)	Disease (n = 45)	*P*-value
Gender	F = 21, M = 24	F = 20, M = 25	1
Age, y	39.42±8.95	40.64±12.00	0.37
BMI	22.38±4.01	22.03±3.20	0.97
PASI	n/a	10.11±7.46	<0.001

Values are presented as the means±standard deviation. *P*-value was calculated by unpaired-Wilcoxon test. There is no PASI score for the control group (n/a).

## Analyses

### Profiling of features from psoriasis and healthy groups

**Figure 1:**
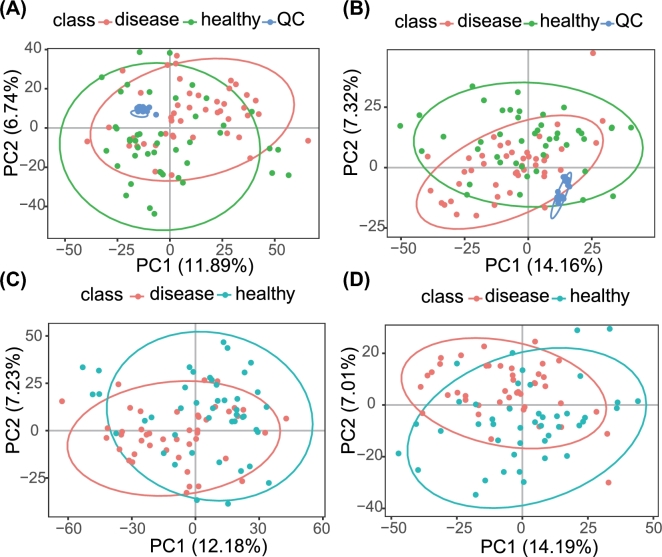
PCA score plots. Overview of PCA score plots obtained from all psoriasis (red), all healthy (green), and QC (blue) samples in positive mode (**A**) and negative mode (**B**). The PCA score map was derived from UPLC-QTOFMS spectra concerning psoriasis (red) and healthy (cyan) samples in positive mode (**C**) and negative mode (**D**).

We detected 11 927 and 5791 features in positive and negative modes, respectively. The numbers of features in QC samples with CVs ≤ 30% were 8428 in positive mode and 4510 in negative mode, with percentages of 70.66% and 77.88%, respectively. After data clean processing, 7817 and 4333 features remained in positive mode and negative modes, respectively. Six hundred eleven (7.25%) and 177 (3.92%) noise features were removed from positive mode and negative mode in data clean processing, respectively. Principal component analysis (PCA) with QC samples was performed to assess the experiment quality. The PCA showed that the pooled QC samples were clustered together in both ion models (positive and negative) (Fig. 1A and B), indicating that the LC-MS analysis process met the required qualifications [19].

**Figure 2:**
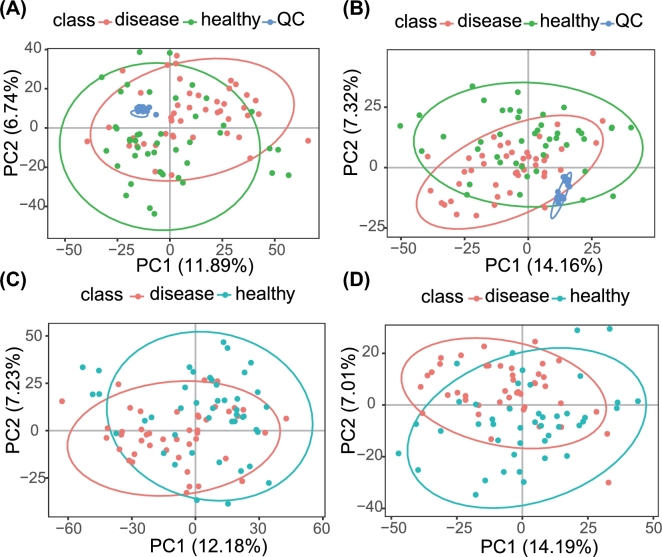
PLS-DA score plots from the healthy and psoriasis groups in (**A**) positive mode (*R*^2^ = 0.699, *Q*^2^ = 0.536) and (**B**) negative mode (*R*^2^ = 0.676, *Q*^2^ = 0.462). Validation plots were obtained from 200 permutation tests in (**C**) positive mode and (**D**) negative mode.

For the statistical analysis, we first applied the PCA to evaluate the separation between the healthy subjects and participants with psoriasis, but the unsupervised multivariate analysis revealed no significant differences between the 2 groups (Fig. 1C, D). To further search for features that may discriminate the 2 groups, a partial least squares discriminant analysis (PLS-DA), which is a supervised multivariate data analysis method, was established testing for differences between features with *P*-values < 0.05. The PLS-DA model clearly distinguished the experimental and control groups based on the lipid dataset (Fig. 2A and B). The model was assessed by monitoring the model goodness of fit (*R*^2^) and predictive ability (*Q*^2^) values, and 200 permutation tests were performed on *R*^2^ and *Q*^2^ as shown in Fig. 2C (positive, *R*^2^ = 0.699, *Q*^2^ = 0.536) and D (negative, *R*^2^ = 0.676, *Q*^2^ = 0.462). A plot of PCA and PLS-DA scores was drawn with the first 2 PCs. Variable importance for projection (VIP) reflects the importance of the variables in the PLS-DA model and was applied to select the important variables. The unpaired Wilcoxon test and Benjamini-Hochberg correction method were also performed for significantly different variable selection. Based on the PLS-DA analysis and *Q*-value evaluation, the criteria of VIP ≥ 1 and *Q*-value < 0.05 were set to discover significant differential features (339 in positive mode and 188 in negative mode) between the psoriasis group and healthy subjects. In total, there were 527 significant features satisfying the criterion.

**Figure 3:**
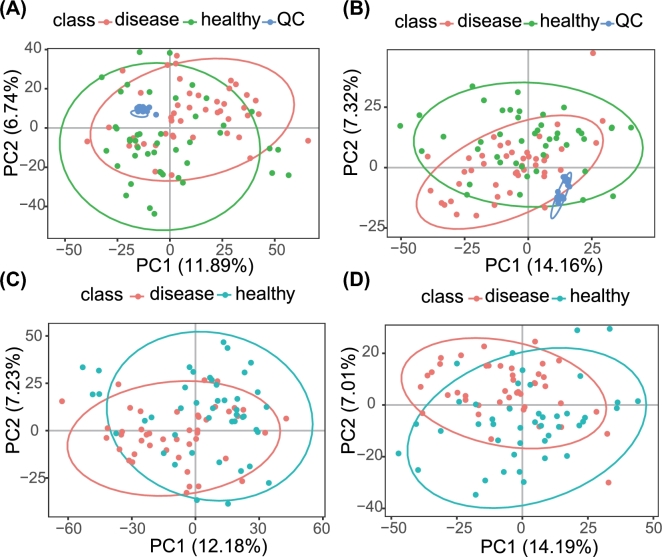
Heatmap of the 17 significantly altered lipids (20 features). (+) represents positive mode, and (-) represents negative mode. The color is proportional to the intensity of change in metabolites; red indicates upregulation, and green indicates down-regulation.

**Figure 4:**
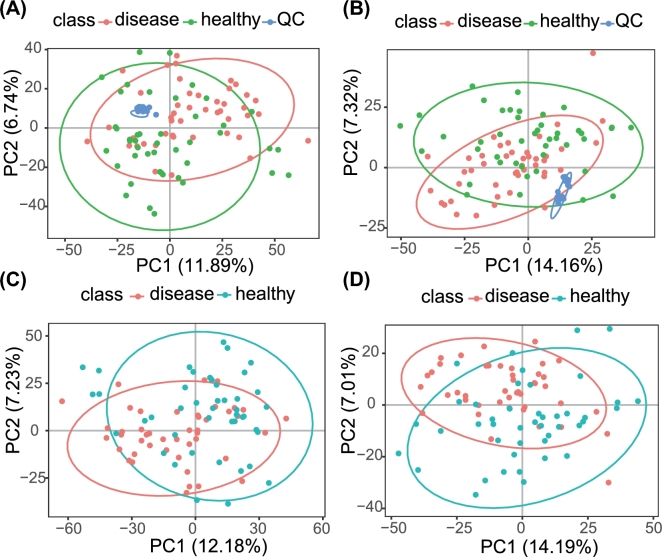
Boxplot of the 17 significantly altered lipids (20 features). Red represents the diseased state, and cyan represents the healthy state. The y-axis is the normalized intensity after log2 transformation. (+) represents positive mode, and (-) represents negative mode. (**A**) LysoPCs, (**B**) PAs, (**C**) PCs, (**D**) PIs.

For those differential features, theoretical database searching and manual spectrum confirmation with specific fragment pattern for different lipid classes were used for identification. Considering the elution rules for different lipid classes, we filtered the theoretical identifications by retention time. Based on the specific fragment patterns and retention times, we ultimately identified 17 lipids (20 features) that were differentially expressed between the healthy and disease groups (Table 2). Among the 17 metabolites, the identification level of LysoPC, PC, and PI was level 1, and the identification level of PA was level 2 [20]. LysoPC (16:0), LysoPC (18:0), and PC (18:0/18:1) were detected in both positive and negative mode, and the abundance pattern in the 2 scan modes was consistent. We generated a heat map as a graphical representation of the differential expression of each lipid (Fig. 3). The result showed that LysoPC and PA were up-regulated in disease group, while PC and PI were down-regulated in the disease group. The relative intensity of changes of those lipids in the 2 groups was also shown in boxplots (Fig. 4; Additional file 2).

**Table 2:** Differential lipids between healthy group and disease group

m/z	ID	mode	Adducts	Neutral mass, Da	Retention time, min	Ratio, disease/ healthy	*P*-value	*Q*-value	VIP	AUC	CI	Formula	Score	Fragmentation Score	Mass Error (ppm)	Isotope Similarity	Description	Identification Level
496.344269	1.32_495.3370n	positive	M+H	495.332490	1.32	1.10	0.0001141	0.0077	1.03	0.73	(0.62–0.83)	C24H50NO7P	51.9	73.8	9.09	95.91	LysoPC(16:0)	level 1
480.309177	1.32_480.3092m/z	negative	M-CH3	495.332490	1.32	1.17	1.43E-05	0.0024	1.25	0.76	(0.66–0.86)	C24H50NO7P	58.2	94.5	−0.63	97.25	LysoPC(16:0)	level 1
510.356220	1.54_509.3486n	positive	M+H	509.348140	1.54	1.25	2.70E-05	0.0033	1.16	0.75	(0.65–0.85)	C25H52NO7P	55.6	81.3	0.82	97.63	LysoPC(17:0)	level 1
524.373115	1.82_523.3658n	positive	M+H	523.363790	1.82	1.16	0.0028	0.0394	1.42	0.68	(0.58–0.78)	C26H54NO7P	56.9	89.5	3.91	99.37	LysoPC(18:0)	level 1
508.340294	1.83_508.3403m/z	negative	M-CH3	523.363790	1.83	1.18	0.0003	0.0108	1.36	0.72	(0.60–0.82)	C26H54NO7P	58.1	95.3	−0.95	96.24	LysoPC(18:0)	level 1
568.341194	1.02_567.3333n	positive	M+H	567.332490	1.02	1.26	0.0001	0.0072	1.13	0.73	(0.62–0.83)	C30H50NO7P	53.8	71.9	1.42	98.81	LysoPC(22:6)	level 1
744.554850	7.00_744.5549m/z	negative	M-CH3	759.577805	7.00	0.80	1.39E-06	0.0008	1.09	0.79	(0.69–0.87)	C42H82NO8P	52.8	66.7	0.06	97.27	PC(16:0/18:1)	level 1
788.618010	7.30_788.6180m/z	positive	M+H	787.609105	7.30	0.76	0.0007	0.0177	1.08	0.71	(0.61–0.80)	C44H86NO8P	43.4	21.4	2.07	98.29	PC(18:0/18:1)	level 1
772.587391	7.29_772.5874m/z	negative	M-CH3	787.609105	7.29	0.80	6.06E-05	0.0046	1.03	0.74	(0.63–0.85)	C44H86NO8P	40.5	6.67	1.63	97.67	PC(18:0/18:1)	level 1
747.498071	5.93_747.4981m/z	negative	M-H	748.505971	5.93	1.62	3.11E-05	0.0037	1.94	0.75	(0.64–0.86)	C43H73O8P	37.9	0	1.39	90.95	PA(40:6)	level 2
723.495919	6.34_723.4959m/z	negative	M-H	724.503819	6.34	1.33	0.0001	0.0075	2.39	0.73	(0.61–0.83)	C41H73O8P	41.5	12.9	−1.53	96.30	PA(38:4)	level 2
809.516853	5.84_809.5169m/z	negative	M-H	810.525829	5.84	0.55	4.22E-06	0.0014	1.82	0.78	(0.67–0.87)	C41H79O13P	37.6	0.618	−2.10	89.97	PI(16:0/16:0)	level 1
807.502829	4.77_807.5028m/z	negative	M-H	808.510179	4.77	0.37	7.34E-05	0.0050	2.02	0.74	(0.64–0.83)	C41H77O13P	37.3	0	−0.09	86.40	PI(16:0/16:1)	level 1
835.533540	6.04_835.5335m/z	negative	M-H	836.541479	6.04	0.71	2.65E-05	0.0035	1.19	0.75	(0.65–0.85)	C43H81O13P	40.6	8.54	−0.79	95.22	PI(16:0/18:1)	level 1
857.518321	4.86_857.5183m/z	negative	M-H	858.525829	4.86	0.71	0.0015	0.0311	1.16	0.69	(0.58–0.80)	C45H79O13P	57	87.6	−0.27	97.98	PI(16:0/20:4)	level 1
889.580608	7.01_889.5806m/z	negative	M-H	890.588429	7.01	0.66	6.58E-06	0.0020	1.33	0.77	(0.66–0.87)	C47H87O13P	39.3	2.78	−0.61	94.64	PI(18:0/20:2)	level 1
887.565099	6.77_887.5651m/z	negative	M-H	888.572779	6.77	0.67	2.16E-05	0.0031	1.37	0.76	(0.65–0.84)	C47H85O13P	46.1	32.7	−0.46	98.21	PI(18:0/20:3)	level 1
913.580432	6.90_913.5804m/z	negative	M-H	914.588429	6.90	0.72	3.65E-05	0.0038	1.24	0.75	(0.65–0.86)	C49H87O13P	39.5	0.347	−0.79	97.93	PI(18:0/22:4)	level 1
859.532505	5.35_859.5325m/z	negative	M-H	860.541479	5.35	0.53	5.40E-05	0.0045	1.61	0.74	(0.64–0.84)	C45H81O13P	37.4	0	−1.97	89.18	PI(18:1/18:2)	level 1
885.548067	5.82_885.5481m/z	negative	M-H	886.557129	5.82	0.46	8.55E-06	0.0021	1.85	0.77	(0.65–0.87)	C47H83O13P	37.2	0.00788	−2.01	88.23	PI(18:1/20:3)	level 1

Seventeen lipids (20 features) were significantly altered and identified. AUC: AUC of the univariate ROC analysis; CI: confidence interval of univariate ROC analysis; fragmentation score: calculated by progenesis QI with theoretical Fragmentation matches; isotope similarity: calculated by progenesis QI with theoretical isotope distribution; score: composite score of mass error, fragmentation score, and isotope similarity.

**Figure 5:**
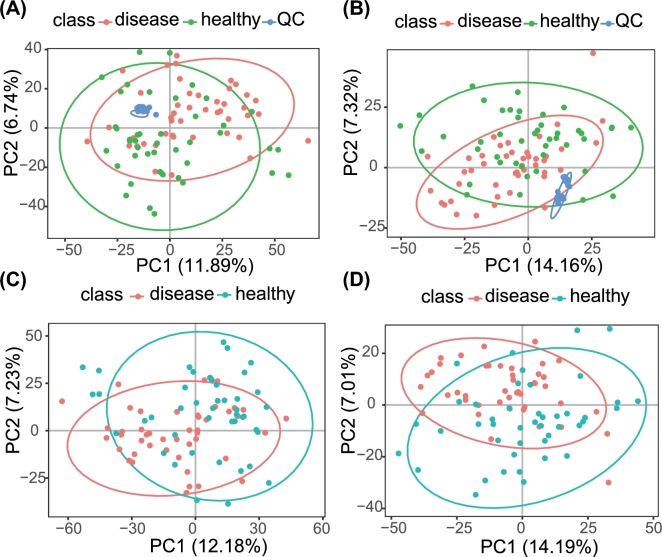
(**A**) ROC curves of LysoPCs with AUC = 0.743, PCs with AUC = 0.747, PAs with AUC = 0.778, and PIs with AUC = 0.758. (**B**) The best combination of metabolites selected from the 17 metabolites using the random forest method (AUC = 0.939). CI: confidence interval.

**Figure 6:**
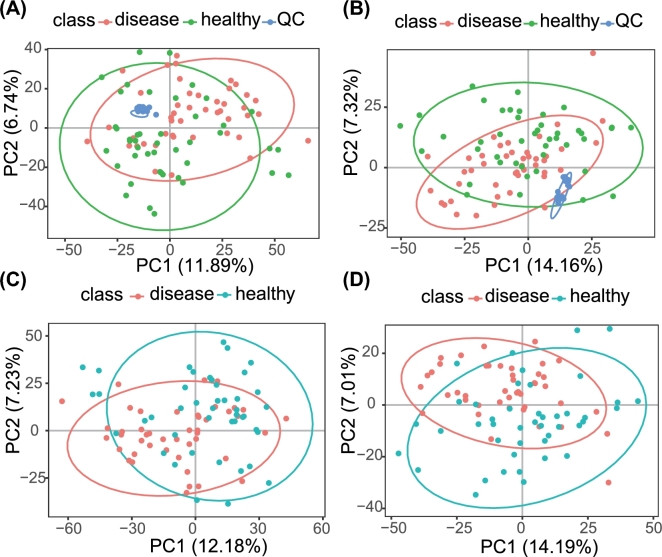
(**A**) The pathway impact plot based on 17 differential lipids using MetaboAnalyst 3.0. Redder colors represent lower *P*-values, and larger circles represent higher impact factors. Low *P*-values and large pathway impact factors indicate that the pathway is greatly influenced. The pathways were mainly enriched in glycerophospholipid metabolism and glycosylphosphatidylinositol (GPI)-anchor biosynthesis. The concentrations of LPA and PA detected in ELISA are shown as (**B**) and (**C**). The asterisk (**) indicates a significant difference (p < 0.01) Student's t test.

We further examined the discrimination of several classes of lipids using multivariate receiver operating characteristic (ROC) curve analysis. The ROC results (Fig. 5A) showed that the area under the curve (AUC) of the LysoPC combination was 0.743, the AUC of the PC combination was 0.747, the AUC of the PA combination was 0.778, and the AUC of the PI combination was 0.758. Ten metabolites from those differentially expressed metabolites were selected by the random forest method, and the AUC of ROC reached up to 0.939 (Fig. 5B). Pathway analyses were performed using MetaboAnalyst [21], and the results (Fig. 6A) showed that metabolites in glycerophospholipid metabolism and glycosylphosphatidylinositol (GPI)-anchor biosynthesis were altered in the disease group.

As described above, we found some differential lipids between the healthy and psoriasis groups. To further explore whether some lipids could distinguish patients with mild or moderate-severe cases from healthy participants, we divided the psoriatic cohorts into mild (n = 25) and moderate-severe (n = 20) groups based on the psoriasis area severity index (PASI), with PASI ≤ 10 representing mild psoriasis and PASI > 10 representing moderate-severe psoriasis. The PCA score plots showed no clear differences among the healthy, mild, and moderate-severe groups (data not shown). The PLS-DA model (data not shown) effectively differentiated between the healthy and mild (*R*^2^ = 0.813, *Q*^2^ = 0.321) and the healthy and moderate-severe (*R*^2^ = 0.820, *Q*^2^ = 0.344) groups; however, it could not differentiate between the mild and moderate-severe groups (*Q*^2^ = –0.135). In addition, after applying the criteria above, there were no differential ions between the mild and moderate-severe groups (data not shown). This revealed that there were no significant differences in lipids among subgroups.

### Lipids identification

Metabolite identification was performed using Progenesis QI (Waters, Nonlinear Dynamics, Newcastle, UK). The LipidMaps [12] and Human Metabolome databases (version 3.6) [18] were used for MS1 identification, and theoretical fragments were used for MS/MS identification. The mass tolerance in MS1 and MS2 was 10 ppm. The elution time of the different lipid classes in the CSH column was also considered for annotation of features according to Additional files 3–4 [22, 23]. Based on these potential identifications, targeted DDA spectra of the significantly different features were acquired for further structure confirmation, as presented in Additional file 5. A standard of LysoPC (18:0) was purchased, and the MS/MS spectrum was collected for metabolite validation (Additional file 6).

### Measurement of LPA and PA concentrations in plasma using ELISA

Because the PA identification rate in the lipid profiling was relatively low, we did not get any differential PAs. However, considering that the LPCs and PCs have important relationships with LPAs and PAs [24], we performed an ELISA to examine LPA and PA abundance in plasma from psoriasis and healthy patients. The results in Fig. 6B and C, show that LPA and PA are dramatically increased in psoriasis patients compared to healthy controls.

## Discussion

Lipids not only comprise the bulk of the cellular membrane bilayer but also regulate a variety of biological processes such as cell proliferation, apoptosis, immunity, angiogenesis, and inflammation [10, 25, 26]. Lipid dysregulation is a pathogenic characteristic of many diseases, including cardiovascular diseases, hypertension, diabetes, and Alzheimer's disease; thus, some dysregulated lipids may act as important biomarkers [27–29]. Lipidomics is an emerging technique for comprehensively analyzing the end products of lipid metabolism and revealing internal changes within whole organisms. Investigation of the lipid byproducts produced by genes or proteins provides clues for understanding cellular regulatory processes and the underlying molecular networks. Because lipids are the end products and the most downstream representation of cellular processes, lipidomics will enable us to gain valuable information regarding the physiology of a system by measuring the amplified output that results from genetic and environmental interactions.

Previous studies showed that lipid metabolites including TC, LDL-C, HDL-C, and ApoA-I are abnormal in psoriatic serum [30] and that fatty acid composition profiles of certain CER subclasses of the SC were significantly altered in epidermal psoriatic patients [31]. In this study, we found that lysoglycerophospholipids, such as LPA and LPC, and glycerophospholipid metabolism, including PA, PC, and PI, were significantly altered in plasma from patients with psoriasis (Figs 4 and 6B and C). LPC and LPA are the most prominent lysoglycerophospholipids and are considered to be inflammatory lipids involved in several immune-mediated diseases such as atherosclerosis and the autoimmune disease systemic lupus erythematosus (SLE) [32–35]. In our study, we found that LPC is significantly increased in psoriasis plasma and that PC shows the opposite. LPC is a type of bioactive lysoglycerophospholipid with high circulating body concentrations (approximately 120 μM) and is a mixture of different components including 16:0 (40%), 18:2 (20%), and 18:1/18:0 (10–15%) [36–38]. LPC is derived from phosphatidylcholine (PC) in lipoproteins or from cell membrane–derived PC in the phospholipase A2 (PLA2) enzyme superfamily via hydrolysis of the sn-2 position fatty acid of membrane PC [38].

Accumulating evidence shows that LPC is raised in inflammation-associated diseases including psoriasis [39] and that LPC exerts its effects through different signaling pathways such as NF-kB, PKC, and ERK in several cell types such as T-lymphocytes, monocytes, and neutrophils. For example, LPC can induce expression of cyclooxygenase type 2 (COX-2), a key pro-inflammatory mediator, via the p38/CREB or ATF-1 pathways in vascular endothelial cells [40, 41]. COX-2 is well known to catalyze arachidonic acid to various classes of bioactive pro-inflammatory lipids such as thromboxanes and prostaglandins, which provides additional clues about the role of lysoglycerophospholipids in inflammatory responses. Interestingly, LPA has also been demonstrated to stimulate COX-2 expression in stromal COX-2. Recently, studies have indicated that LPC could be a high-affinity ligand for G2A that triggers immune-related signaling pathways [42, 43]. G2A is a type of G protein–coupled receptor that is expressed in immunoregulatory cell types such as neutrophils, T cells, and macrophages [44, 45].

LPA is also increased in psoriatic plasma, as shown in Fig. 6B. LPA is a bio-activated lipid that has been detected in various fluids such as serum, seminal fluid, and follicular fluid. Compared with LPC, the total plasma LPA concentration is much lower than LPC [38]. LPA has multiple functions in almost all mammalian cell types such as endothelial cells, T lymphocytes, and dendritic cells, which are dependent on the LPA receptor and G-protein-coupled receptor classified from LPA1–LPA6 [46–48]. Notably, the transcription factor peroxisome proliferator–activated receptor γ (PPAR-γ) was identified as an intracellular receptor for LPA [49, 50]. LPA initiates signaling pathways or exerts biological effects through different receptor subtypes; for example, LPA promoted cell growth and differentiation through the LPA receptor 1,3 and PPAR-γ, which facilitated hyperplasia during inflammation in mast cells [24, 51].

PA is not only a major constituent of the cell membrane but also a biosynthetic precursor for the formation (directly or indirectly) of all cellular acylglycerol lipids. The conversion of PA into DAG by lipid phosphate phosphohydrolases is a critical step for the production of PC. In addition, DAG can be converted into cytidine diphosphate (CDP)–DAG, which is a precursor for phosphatidylglycerol (PG) and protease inhibitors (PIs). PA also acts as a secondary messenger to mediate downstream signaling pathways such as the mTOR pathway [52–55]. Synthesis via the glycerophospholipid pathway, named the Kennedy pathway [56–58], was elucidated in the early 1960s. PA is synthesized in several steps from glycerol-3-phosphate, which is derived from glycolysis or the phosphorylation of glycerol and fatty acetyl coenzyme A by enzymes such as glycerol-3-phosphate acyltransferases (GPAT); therefore, PA is considered a critical product of glycolysis and glycerophospholipid metabolism. PA can be converted into DAG or CDP-DAG by CDP-DAG synthase, which are phospholipid biosynthesis precursors. CDP-alcohol phosphotransferase enzymes such as choline/ethanolamine phosphotransferase (CEPT) and choline phosphotransferase have been demonstrated to be indispensable in the biosynthesis of PC, which catalyzes the formation of a phosphodiester bond linking the head and tail components of the lipid.

In conclusion, we employed plasma lipidomics to investigate the potential pathophysiology of psoriasis. The profiles, including lysoglycerophospholipids such as LPC and LPA and glycerophospholipids such as PA, PC, and PI, are dramatically altered in psoriasis plasma. However, mechanistic studies will be required to explore the details and distinct biochemical characteristics and the cellular effects of lipid species on both T cell and keratinocyte responses in the pathogenesis of psoriasis.

### Potential implications

Psoriasis is a chronic, systemic inflammation disease consequence of the interactions between genetic and environmental factors. IL-23/IL-17/IL-22 axes produced by abnormal activation of Th17 cells play key roles in pathogenesis of psoriasis that stimulate keratinocyte proliferation and secretion of other inflammatory cytokines such as TNF-α, IL-1, IL-6, and IL-8. Currently, metabolism and immunity is a hotspot, particularly in Th17 cell activation. Accumulating evidence has demonstrated that glycolysis regulated by mTOR and the HIF-1α signaling pathway facilitates Th17 cell differentiation and inhibition of HIF-1α and mTOR activation from CD4+ T cells; treatment with 2-DG attenuates Th17 differentiation through inhibition of glycolysis. Although we found that glycerophospholipids metabolism is dysfunction in psoriasis, the details of the mechanism are lacking, especially for Th17 cell differentiation; therefore, the elaboration of the relationship between glycerophospholipid metabolism and Th17 activation is an important direction for the future in the pathogenesis of psoriasis.

## Methods

### Sample collection

Healthy controls (n = 45) and patients with mild or severe psoriasis (n = 45) were recruited at the Xiangya Hospital Central South University in accordance with the Declaration of Helsinki. All sample donors provided signed consent forms, which were approved by the Xiangya Hospital Committee of Ethics. Mild and moderate-severe psoriasis patients (defined as PASI > 10) were recruited from patient pools without systemic therapy. None of the patients were on prescribed anti-inflammatory drugs. All samples were obtained prior to the commencement of any treatment. The recruited participants consisted of 90 age- and gender-balanced individuals (45 healthy controls, 20 mild and 25 moderate-severe psoriasis patients) (Additional file 1). For analysis purposes, the participants were subdivided into healthy and disease groups (n = 45 each), referred to as the exploratory and validation subjects. After fasting overnight, 10 mL of whole blood was collected from each subject into ethylene diamine tetraacetic acid (EDTA) tubes. Samples were left standing for 1 hour before centrifugation at room temperature for 20 minutes at 3100 g. After centrifugation, plasma samples were collected and immediately stored at –80°C until use. Psoriasis was judged as severe according to the PASI, which is an established measurement that quantifies the thickness, redness, scaling, and distribution of psoriasis lesions. This study was approved by the Regional Committee of Ethics.

### Sample preparation and lipid extraction

Prior to the experiment, samples were left at –20°C for 30 minutes and then thawed at 4°C until no ice was observed in the tubes. The lipid extraction method followed a previously published paper [22]. Briefly, 40 μL of plasma was extracted with 120 μL precooled isopropanol (IPA) then vortexed for 1 minute, and after incubation for 10 minutes at room temperature, the mixture was stored overnight in the refrigerator at –20°C to improve protein precipitation. Samples were centrifuged for 20 minutes at 14 000 g, and then the supernatant was further diluted with IPA/acetonitrile (ACN)/H_2_O (2:1:1 v:v:v) and stored at –80°C until LC-MS analysis. Equal amounts of all samples were pooled as a QC sample for LC-MS system conditioning and quality control [59].

### The UPLC-MS/MS method

Lipidomics was performed on an ACQUITY UPLC system (Waters, Manchester, UK) coupled with a G2-XS QTOF mass spectrometer (Waters, Manchester, UK). Chromatographic separation was employed with an ACQUITY UPLC CSH C18 column (2.1 × 100 mm, 1.7 μm, Waters). Mobile phase A consisted of 10 mM of ammonium formate and 0.1% formic acid (ACN: H_2_O = 60:40, v/v), and mobile phase B consisted of 10 mM of ammonium formate and 0.1% formic acid (IPA: ACN = 90:10, v/v). A flow rate of 0.4 mL/min was used. The initial elution was started at 40% B and was immediately increased by a linear gradient to 43% B for the first 2 minutes, followed by an increase to 50% B within 0.1 minutes. Over the next 3.9 minutes, the gradient was increased to 54% B, and the amount of B was increased to 70% during the next 0.1 minutes. In the final part of the gradient, B was increased to 99% and maintained for 1.9 minutes. Finally, B was returned to 40% over the next 0.1 minutes and equilibrated for 1.9 minutes for the next injection. Both positive and negative modes were performed and operated in Centroid MS^E^ mode with an acquisition time of 0.2 seconds per scan. The scan range was set at 50–1800 Da. The capillary was set at 0.25 kV and 2 kV in positive ion mode and negative ion mode, respectively. Sampling cone voltages were set at 40 V in both modes. The source temperature was set to 120°C. The desolvation temperature and gas flow were 500°C and 800 L/h. Leucine enkephalin (MW = 555.62) was applied as a lock mass for accurate mass measurements, and sodium formate solution was used for mass calibration. Furthermore, QC samples were interspersed in samples to evaluate the stability of the LC-MS system during acquisition (Additional file 7).

### ELISA analysis of plasma LPA and PA

The plasma derived from age- and gender-matched psoriasis patients (*n* = 25) and healthy individuals (*n* = 25) was prepared as previously described. The ELISA kit for testing LPA and PA was obtained from Shanghai Xinyu Biotechnology Co. Ltd. (Shang Hai, China). The experimental procedure follows ELISA protocol.

### Data processing

The raw files were imported into Progenesis QI software for peak alignment and picking. Data generated from Progenesis QI (Additional file 8) were further preprocessed using metaX software [60]. Features were removed from further analysis if they were detected in less than 50% of the QC samples or less than 20% of the experimental samples. After the previous filtering, missing values were imputed using the k-nearest neighbor method. The QC-robust spline batch correction (QC-RSC) [59] and Combat normalization methods [61] were used to correct signal drift and batch variation. After normalization, features with a relative standard deviation of less than 30% in the QC samples were retained. Prior to statistical analysis, data clean algorithms were applied to the dataset. Features were removed if SNR < 1 (SNR = standard deviation_sample_/standard deviation_QC_) or the relative difference between the mean QC samples intensity compared to the mean study sample intensity was more than 3 times the standard deviation of the study sample intensity.

### Statistical analysis

Multivariate and univariate analyses were also conducted using metaX [60]. A PCA was performed to detect outliers, and a PLS-DA [62] was applied using log transformation and Pareto scaling. Permutation testing (200 times) on the *R*^2^ and *Q*^2^ of the PLS-DA was used to assess the reliability of the PLS-DA model [63]. The unpaired-Wilcoxon test was performed to test significant differences between the control and experimental groups, and the *P*-value was adjusted for multiple hypothesis testing using the Benjamini-Hochberg method.

The univariate and multivariate receiver operating characteristic curve was applied to detect potential biomarkers. To create the classification model between the experimental and control groups, functions implemented in metaX [60] were used for biomarker selection, model creation, and performance evaluation. In short, the best feature set for classification was evaluated and used to build a random forest model. In all, 2/3 of the subjects were selected randomly as a training set, and the rest were used as a test set. To prevent overfitting in the training set, a 7-fold cross-validation was applied in the random forest modeling.

## Availability of source code and requirements

Project name: Psoriasis

Project home page: https://github.com/ZengVera/psoriasis

Operating system(s): platform independent

Programming language: R

Other requirements: R 3.2.0 or higher, metaX package

License: GNU General Public License version 2.0 (GPLv2).

Any restrictions to use by non-academics: none

## Availability of data materials

Raw data (MS^E^) for all samples including QC samples reported here are available at the MetaboLights database (MetaboLights, RRID:SCR_014663) with the accession number MTBLS408. The code we performed can be acquired at https://github.com/ZengVera/psoriasis [64]. Further supporting data and snapshots of our code in GitHub are available in the *GigaScience* repository, *Giga*DB (*Giga*DB, RRID:SCR_004002) [65]. Further details on experimental protocols can be found in Zeng et al. [66].

## Additional files

Additional file 1: Phenotype of 90 enrolled subjects.

Additional file 2: Boxplot of differentially expressed lipid.

Additional file 3: Lipid retention time range in positive mode.

Additional file 4: Lipid retention time range in negative mode.

Additional file 5: Targeted DDA MS/MS spectrum or MS/MS spectrum extracted by Progenesis QI software from MS^E^ raw data.

Additional file 6: MS/MS spectrum of standard LysoPC (18:0).

Additional file 7: Run order of samples and QCs in LC-MS analysis.

Additional file 8: Table of peak intensity generated by Progenesis QI software.

## Abbreviations

ACN: acetonitrile; DAG: diacylglycerol; DDA: Data-Dependent Acquisition; EDTA: ethylene diamine tetraacetic acid; IPA: isopropanol; LPA: lysophosphatidic acid; LPC: lysophosphatidylcholine; PA: phosphatidic acid; PASI: psoriasis area severity index; PC: phosphatidylcholine; PCA: principal component analysis; PI: phosphatidylinositol; PLS-DA: partial least squares discriminant analysis; QC: quality control; QC-RSC: QC-robust spline batch correction; SNR: Signal to Noise Ratio; VIP: variable importance for projection.

## Consent for publication

The study was approved by the ethics committee of Xiang Ya Hospital, Central South University. Written informed consent was obtained from all patients prior to sampling.

## Competing interests

None of the authors have potential conflicts of interest to disclose.

## Funding

This work was supported by Grant No. 81430075 from the Key Project of the National Science Foundation, Grant No. 81572679 from the National Natural Science Foundation, and Grant No. 2015JJ2161 from the Natural Science Foundation of Hunan province.

## Author contributions

Conception and design: Cong Peng, Xiang Chen, Bo Wen. Sample collection: Xuekun Jia, Wu Zhu, Jie Li, Yanhong Kuang, Weiqi Zeng. Experiment: Guixue Hou, Xiaomin Chen, Lei Li. Data analysis: Chunwei Zeng, Bo Wen, Zhanlong Mei, Guixue Hou. Manuscript writing: Cong Peng, Chunwei Zeng, Bo Wen. Siqi Liu participated in discussions. All authors reviewed this manuscript.
